# Reduced USP39 expression inhibits malignant proliferation of medullary thyroid carcinoma in vitro

**DOI:** 10.1186/s12957-015-0669-4

**Published:** 2015-08-25

**Authors:** Yong An, Shuwen Yang, Kai Guo, Ben Ma, Yu Wang

**Affiliations:** Department of Head & Neck Surgery, Fudan University Shanghai Cancer Center and Department of Oncology, Shanghai Medical College, Fudan University, 6W of No.3 Building, No.270 DongAn Road, Shanghai, 200223 China

**Keywords:** Medullary thyroid carcinoma, Ubiquitin-specific peptidase 39, shRNA, Targeted therapy

## Abstract

**Background:**

Medullary thyroid carcinoma (MTC) constitutes approximately 5 % of all thyroid cancers and carries a worse prognosis than other differentiated thyroid cancers. Targeted therapies are being investigated for systemic treatment of MTC. Ubiquitin-specific peptidase 39 (USP39) functions in pre-mRNA splicing as a component of the U4/U6-U5 tri-snRNP and also participates in spindle checkpoint and cytokinesis. In this study, we aimed to evaluate the potential role in MTC.

**Methods:**

We used lentivirus-delivered short hairpin RNA (shRNA) to silence USP39 expression in one MTC cell line TT. USP39 expression was detected by qPCR and Western blot. For functional analysis, MTT assay was performed to evaluate the proliferation activity, and FACS was used to assess the cell distribution in the cell cycle. Moreover, the expressions of cell cycle-related proteins were examined by Western blot.

**Results:**

Both two shRNA sequences against USP39 could efficiently reduce its expression in TT cells. Knockdown of USP39 significantly decreased cell proliferation and caused cell cycle arrest at G2/M phase. Moreover, G2/M phase-associated proteins, Cyclin B1 and CDK1, were obviously down-regulated in TT cells after USP39 silencing.

**Conclusions:**

Therefore, knockdown of USP39 is likely to provide a novel alternative to targeted therapy of MTC and deserves further investigation.

## Background

Medullary thyroid carcinoma (MTC) accounts for 5–10 % of all thyroid cancers and carries a worse prognosis than other differentiated thyroid cancers with an overall 10-year survival rate of 75–85 % [1, 2]. MTC is a neuroendocrine tumor originating from the parafollicular C cells of the thyroid gland [3]. Calcitonin and carcinoembryonic antigen are useful blood markers and are often used in patient’s follow-up. Although the majority of MTC cases are sporadic, approximately 20 % are hereditary because of a germline mutation in the rearranged during transfection proto-oncogene (RET) [4–6]. Hereditary MTC can present in isolation (familial medullary thyroid cancer (FMTC)) or as part of the multiple endocrine neoplasia syndrome type 2 (MEN2, MEN2A, or MEN2B). Patients with newly diagnosed disease are treated with surgery. A common discovery in MTCs that is diagnosed late is lymph node metastasis in the upper mediastinum and neck. Metastases occur in ~70 % of patients with MTC who have a palpable thyroid nodule (>1.0 cm diameter) [7]. At this stage of the disease, patients cannot undergo surgical resection and biochemical “cure” rates drop to ≤30 % [8, 9]. Therefore, systemic radiation and chemotherapy therapy are not believed to represent important or useful options for most patients with MTC.

Ubiquitin-specific peptidase 39 (USP39) contains a central zinc finger and two ubiquitin C-terminal hydrolase (UCH) domains. Interestingly, USP39 does not have ubiquitin-specific peptidase activity but plays an essential role in pre-mRNA splicing as a component of the U4/U6-U5 tri-snRNP, one of the building blocks of the spliceosome [10]. Its N-terminal domain is rich in arginines, serines, and glutamic acids and resembles the RS domain of serine/arginine (SR)-related proteins, which is essential to recruit the tri-snRNP to the prespliceosome [11]. USP39 has been implicated in the assembly of the mature spliceosome complex [12]. USP39 is also required to maintain mitotic spindle checkpoint integrity and support successful cytokinesis, probably through splicing of Aurora B and other mRNAs that are essential for proper spindle checkpoint function [13]. Depletion of USP39 by RNAi induced massive polypoidization in U2OS cells, possibly a result of defective chromosome segregation and cytokinesis, suggesting USP39 as a key regulator of mitosis. Recently, some studies have reported the role of USP39 in cancer cell growth. Wang et al. demonstrated that USP39 is expressed at a higher level in breast cancer and regulates cell growth and survival [14]. Wen et al. demonstrated that the overexpression of USP39 could enhance the proliferation of prostate cancer cells, suggesting that USP39 may also play a role in prostate cancer development [15]. However, the involvement of USP39 in MTC remains unclear.

The aim of this study was to explore the potential role of USP39 in MTC cell growth. We carried out loss-of-function analysis in one human MTC cell line TT using lentivirus-delivered short hairpin RNA (shRNA) targeting USP39. After USP39 silencing, cell proliferation was suppressed and cell cycle was arrested at G2/M phase. Therefore, the inhibition of USP39 by shRNA might be a potential therapeutic method in MTC.

## Methods

### Materials

F-12K medium was obtained from Gibco (Carlsbad, CA, USA, cat no.21127022). DMEM was obtained from Hyclone (Logan, UT, USA, cat no.SH30243.01B+). Fetal bovine serum (FBS) was obtained from Biowest (Loire Valley, France, cat no.S1810). Lipofectamine 2000 and TRIzol® Reagent were purchased from Invitrogen (Carlsbad, CA, USA). M-MLV Reverse Transcriptase was purchased from Promega (Madison, WI, USA). All other chemicals were obtained from Sigma-Aldrich (St. Louis, MO, USA). The lentiviral vector (pFH-L) and packaging vectors (pVSVG-I and pCMVΔR8.92) were purchased from Shanghai Hollybio (Shanghai, China). The antibodies used were as follows: rabbit anti-USP39 antibody (1:1000 dilution; Proteintech Group, Inc., cat no.23865-1-AP), p-CDC2/CDK1(Tyr15) antibody (1:1000 dilution; Cell Signaling Technology, cat no.9111), mouse anti-CDC2/CDK1 antibody (1:1000 dilution; Cell Signaling Technology, cat no.9116), rabbit anti-GAPDH antibody (1:100,000 dilution; Proteintech Group, Inc., cat no.10494-1-AP), and horseradish peroxidase-conjugated goat anti-rabbit (1:5000 dilution; Santa Cruz, cat no.SC-2054) secondary antibodies.

### Cell culture

Human MTC cells TT and human embryonic kidney cells 293T were purchased from the Cell Bank of Chinese Academy of Science (Shanghai, China). TT cells were maintained in F-12K medium supplemented with 10 % FBS. Maintained in DMEM supplemented with 10 % FBS were 293T cells. These cell lines were cultured at 37 °C in humidified atmosphere of 5 % CO2.

### Lentivirus-delivered short hairpin RNA transduction

Two shRNA sequences targeting human USP39 gene (NCBI accession number: NM_001256725.1) were 5′- GATTTGGAAGAGGCGAGATAACTCGAGTTATCTCGCCTCTTCCAAATCTTTTT-3′ (S1) and 5′-CCTTCCAGACAACTATGAGATCTCGAGATCTCATAGTTGTCTGGAAGGTTTTT-3′ (S2), which were subjected to BLAST analysis against the human genome database to eliminate cross-silence phenomena with nontarget genes. A scrambled fragment (5′-GCGGAGGGTTTGAAAGAATATCTCGAGATATTCTTTCAAACCCTCCGCTTTTTT-3′) that has no significant homology with mouse or human gene sequences was used as a negative control. The shRNAs were cloned into the pFH-L vector by use of NheI/PacI restriction sites, which was then transfected into 293T cells with packaging vectors (pVSVG-I and pCMVΔR8.92) using Lipofectamine 2000 according to the manufacturer’s instructions. The supernatant was collected 48 h later, centrifuged (4000 *g*, 4 °C, 10 min) to remove cell debris, filtered through 0.45-μm cellulose acetate filters, and then concentrated again (4000 *g*, 4 °C, 15 min). TT cells were dispensed into 6-well plates at a density of 150,000 cells per well and transduced with shRNA-expressing lentivirus (shCon or shUSP39(S1)/(S2)) at a multiplicity of infection (MOI) of 60. The lentiviral vectors expressed green fluorescence protein (GFP), which allowed for measurement of infection efficiency in transduced cells.

### Quantitative real-time PCR analysis

TT cells were harvested after lentivirus transduction for 5 days. Total cellular RNA was extracted using Trizol reagent and reversely transcribed to cDNA by M-MLV reverse transcriptase according to the manufacturer’s instructions. qPCR products were detected with SYBR Green on BioRad Connect Real-Time PCR platform. qPCR procedure was denatured at 95 °C for 1 min, 40 cycles of denaturation at 95 °C for 5 s, and extension at 60 °C for 20 s. β-actin gene was amplified as internal control. Relative quantitation was analyzed by taking the difference ΔC(T) between the C(T) of β-actin and C(T) of target gene and computing 2^−ΔΔC(T)^. The following primers were used: USP39, 5′-GCCAGCAGAAGAAAAAGAGC-3′ (forward) and 5′-GCCATTGAACTTAGCCAGGA-3′ (reverse); CCNB1 (Cyclin B1), 5′-CTGTTGGTTTCTGCTGGGTGTAG-3′ (forward) and 5′-CGCCTGCCATGTTGATCTTCG-3′ (reverse); β-actin, 5′-GTGGACATCCGCAAAGAC-3′ (forward) and 5′-AAAGGGTGTAACGCAACTA-3′ (reverse).

### Western blotting analysis

TT cells were harvested after lentivirus transduction for 5 days. Total protein was extracted with 2× SDS Sample Buffer (100 mM Tris–HCl (pH 6.8), 10 mM EDTA, 4 % SDS, 10 % Glycine). Equal amounts of lysate (30 μg) in each lane, as determined by the bicinchoninic acid (BCA) assay, were separated by 10 % SDS-PAGE and transferred to PVDF membranes. The membranes were blocked with 5 % nonfat dry milk in Tris-buffered saline with Tween 20 (TBST) for 1 h at room temperature and incubated with TBST containing anti-USP39 or anti-GAPDH antibody overnight at 4 °C, followed by incubation with secondary antibody for 1 h at room temperature. The blots were detected using enhanced chemiluminescence (ECL) kit (Amersham) and visualized by exposure to X-ray film. GAPDH was used as a control to verify equal protein loading.

### MTT assay

To evaluate the effect of USP39 on MTC cell proliferation, 3-(4,5-dimethylthiazol-2-yl)-2,5-diphenyl-tetrazolium bromide (MTT) colorimetric assay was performed in TT cells after lentivirus transduction for 4 days. Briefly, TT cells were dispensed into 96-well plates at a concentration of 7000 per well. The plates were incubated for 1 to 5 days at 37 °C. On each day, 20 μL of MTT (5 mg/mL) was added and incubated for 4 h. Afterwards, the entire supernatant was discarded and acidic isopropanol (10 % SDS, 5 % isopropanol and 0.01 mol/L HCl) was added at a volume of 100 μL per well and incubated at 37 °C for 12 h. The absorbance at 595 nm of each well was determined using an ELISA reader.

### Fluorescence-activated cell-sorting analysis

To evaluate the effect of USP39 on MTC cell cycle progression, flow cytometry assay was performed in TT cells after lentivirus transduction for 6 days. Briefly, TT cells were dispensed into 6-cm dishes at a concentration of 200,000 per dish. After culture at 37 °C for 40 h, cells were harvested, fixed in 70 % ethanol, and stored overnight at 4 °C. The cells were then treated with NaCl/Pi staining solution (50 μg/mL PI and 100 μg/mL RNase A). Following incubation for 1 h in the dark at room temperature, cells were analyzed by flow cytometry (FACSCalibur; Becton Dickinson, San Jose, CA, USA). The fractions of the cells in G0/G1, S, and G2/M phases were analyzed with dedicated software (Becton Dickinson).

### Statistical analysis

Statistical analysis was performed using SPSS 16.0 software package (SPSS Inc, Chicago, IL, USA). All data were expressed as mean ± standard deviation (SD). Differences between two groups were analyzed by Student’s *t* test, and a *p* value of less than 0.05 was considered statistically significant.

## Results

### Knockdown of USP39 expression with lentivirus-delivered shRNA

**Fig. 1:**
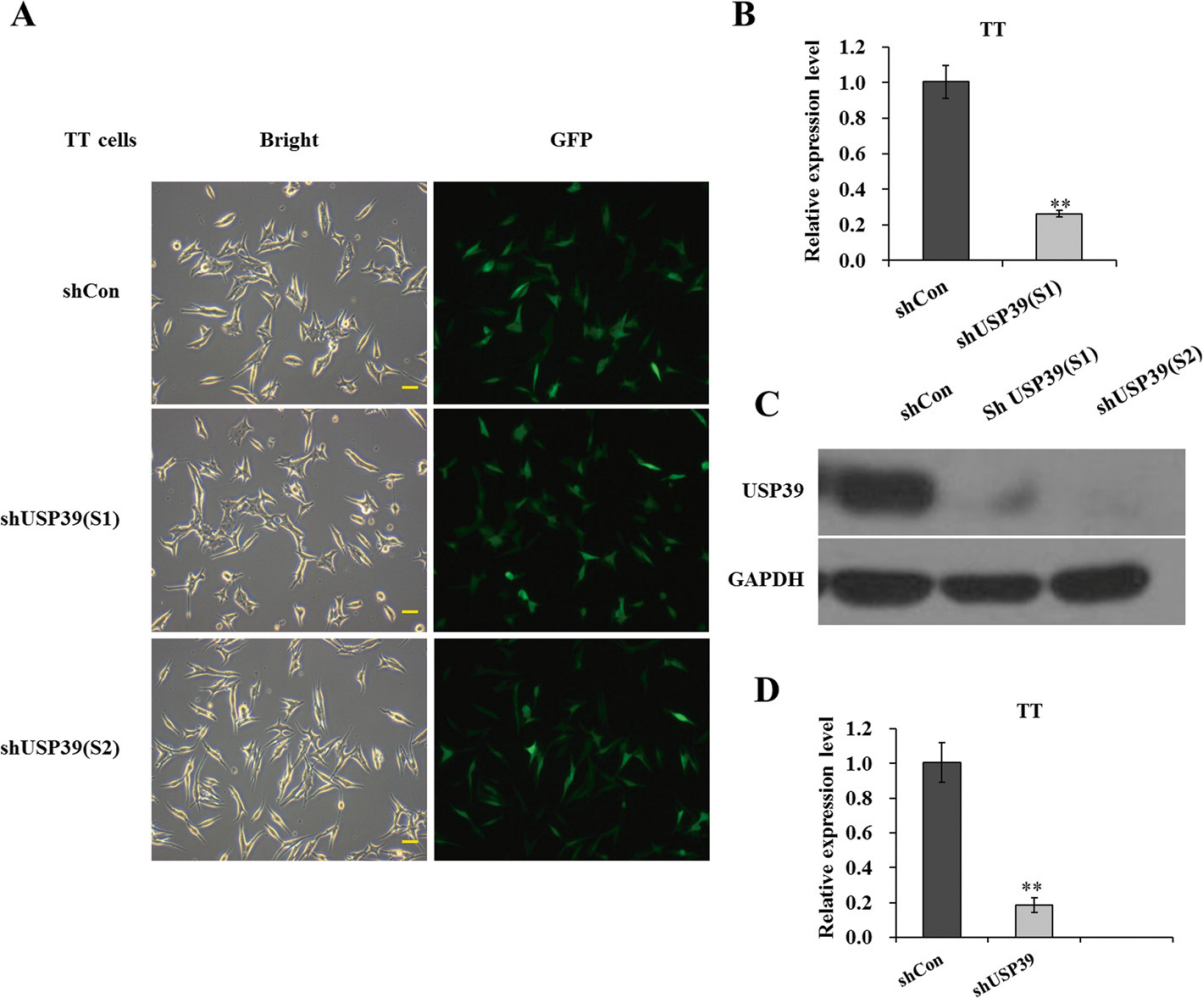
Lentivirus-delivered shRNA targeting USP39 depleted its endogenous expression in TT cells. **a** Evaluation of the lentivirus transduction rate, which was more than 80 % as calculated by cellular enumeration using fluorescence and light microscopy. **b** Quantitative analysis of USP39 knockdown efficiency (S1) in TT cells was assessed by qRT-PCR. β-actin gene was used as an internal control. **c** Representative immunoblot showing USP39 knockdown efficiency determined in TT cells. GAPDH protein was used as an internal control. **d** Quantitative analysis of USP39 knockdown efficiency (S2) in TT cells was assessed by qRT-PCR. β-actin gene was used as an internal control. Each point represents the mean ± SD of three independent repeats. The significance was determined by *t* test. **p < 0.01; scale bar, 10 μm

TT cells were transduced with shRNA-expressing lentivirus (shCon or shUSP39(S1)/(S2)). GFP expression was observed by fluorescent microscopy 4 days post-transduction. As depicted in Fig. 1a, over 80 % of cells expressed GFP in shCon, shUSP39(S1), and shUSP39(S2) groups, indicating a successful infection rate. The inhibitory effect of USP39 shRNA on its endogenous expression in TT cells was examined by qRT-PCR and Western blotting. As depicted in Fig. 1b, the mRNA level of USP39 was significantly reduced in TT cells infected with shUSP39(S1) with a knockdown efficiency of 73.9 %, in contrast to cells infected with shCon. Immunoblot further verified the down-regulation of USP39 expression at protein level (Fig. 1c). The mRNA level of USP39 was also significantly reduced in TT cells infected with shUSP39(S2) in contrast to cells infected with shCon (Fig. 1d). Therefore, lentivirus-delivered shRNA could specifically deplete endogenous USP39 expression in TT cells.

### Effect of USP39 knockdown on cell proliferation and cell cycle progression

**Fig. 2:**
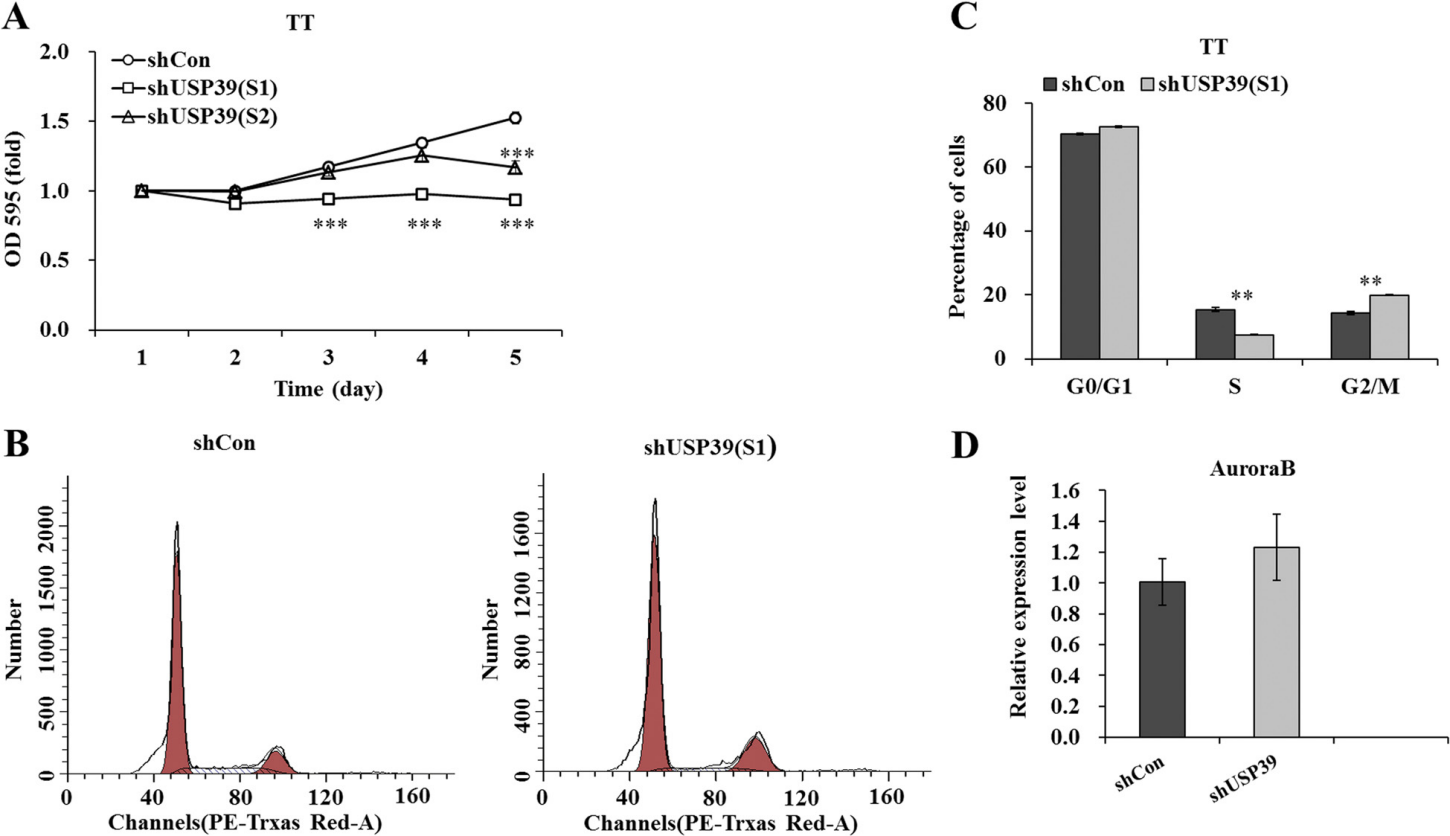
Knockdown of USP39 inhibited proliferation of TT cells. **a** MTT showing growth curves determined in TT cells. The number of viable cells was much fewer in shUSP39(S1)/(S2) groups than in shCon group. **b** Comparison of the cell population in G0/G1, S, and G2/M phase between shCon and shUSP39(S1) groups was assessed by flow cytometry. **c** The percentage of cells in G2/M phase was significantly higher in the shUSP39(S1) group than in the shCon group, while the percentages of cells in S phase was simultaneously reduced. **d** Quantitative analysis of Aurora B expression alteration in TT cells was assessed by qRT-PCR. β-actin gene was used as an internal control. Each point represents the mean ± SD of three independent repeats. The significance was determined by *t* test. **p < 0.01

We next examined the effects of USP39 knockdown on proliferation of TT cells. After infection of USP39 shRNA, MTT assay was performed in TT cells for five consecutive days. As depicted in Fig. 2a, the number of viable cells infected with shUSP39(S1)/(S2) was much fewer than those infected shCon (*p* < 0.001). Besides, the inhibitory effect was much stronger in shUSP39(S1) group than in shUSP39(S2) group. This indicates that knockdown of USP39 could strongly decrease the proliferation of TT cells.

To test the mechanism by which USP39-modulating cell proliferation, the flow cytometry assay was used to determine the cell cycle of TT cells. Representative images of cell cycle distribution were presented as Fig. 2b. As depicted in Fig. 2c, the cell percentage of G2/M phase was increased from 14.29 ± 0.46 % in shCon-infected cells to 19.85 ± 0.12 % in shUSP39(S1)-infected cells. By contrast, the cell percentage of S phase was decreased from 15.37 ± 0.63 % in shCon-infected cells to 7.54 ± 0.16 % in shUSP39(S1)-infected cells. This indicates that USP39 shRNA could strongly block (*p* < 0.01) the cell cycle progression of TT cells. Taken together, these results suggest that knockdown of USP39 could inhibit TT cell proliferation by inducing G2/M phase cell cycle arrest. As depicted in Fig. 2d, there is no significant difference of Aurora B mRNA level between shCon-infected cells and shUSP39(S1)-infected cells.

### Effect of USP39 knockdown on G2/M-related cell cycle regulators

**Fig. 3:**
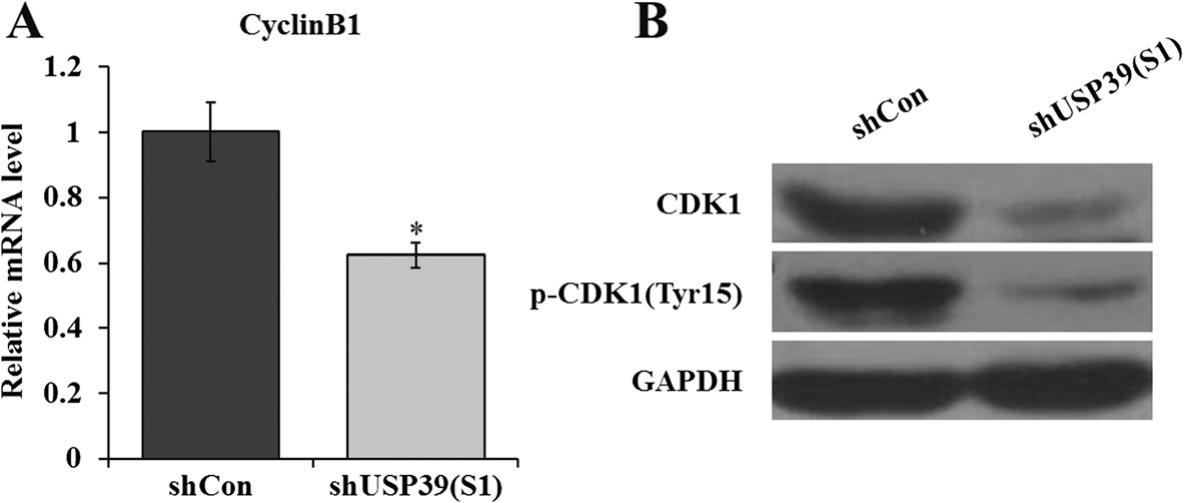
Knockdown of USP39 down-regulates G2/M-related cell cycle regulators. **a** Quantitative analysis of Cyclin B1 expression alteration in TT cells was assessed by qRT-PCR. β-actin gene was used as an internal control. **b** Representative immunoblot showing CDK1 and p-CDK1(Tyr15) protein levels determined in TT cells. GAPDH protein was used as an internal control. Each point represents the mean ± SD of three independent repeats. The significance was determined by *t* test. *p < 0.05

In mammalian cells, G2/M transition is controlled by cyclin-dependent kinase (CDK1) (also known as CDC2), which partners with Cyclin B1. During G2 phase, the CDK1/Cyclin B1 complex is held in an inactive state by CDK1 phosphorylation at two negative regulatory sites, threonine-14 (Thr14) and tyrosine-15 (Tyr15). Therefore, we further examined the effect of USP39 knockdown on the CDK1/Cyclin B1 complex. As depicted in Fig. 3a, the mRNA level of Cyclin B1 was significantly down-regulated in TT cells with the absence of USP39 (*p* < 0.05). Besides, both the phosphorylated and basal levels of CDK1 were reduced by USP39 knockdown (Fig. 3b). These results indicate that knockdown of USP39 induced G2/M phase cell cycle arrest via suppression of the CDK1/Cyclin B1 complex.

## Discussion

Spliceosome factors have been shown to be associated with cancer development. For example, SF3B1 (Splicing factor 3B subunit 1) is highly mutated in various hematological malignancies such as chronic lymphocytic leukemia and myelodysplastic syndromes [16, 17]. In this study, we provided new evidence that USP39, a *bona fide* spliceosome factor, is involved in the proliferation regulation of MTC cells. Lentivirus-delivered short hairpin RNA (shRNA) targeting USP39 was used to stably down-regulate its endogenous expression in MTC cells TT. Knockdown of USP39 significantly inhibited proliferation of TT cells in vitro. It has been shown that USP39 is required to maintain the spindle checkpoint and support successful cytokinesis [13]. Moreover, flow cytometry analysis was performed to test the effect of USP39 knockdown on mitosis. The cell cycle of TT cells was arrested at G2/M phase with the absence of USP39, which could contribute to the inhibition of cell proliferation. Our results were consistent with a previous research showing that knockdown of USP39 markedly reduces the proliferation of MCF-7 breast cancer cells [14], suggesting that USP39 may play a role in cancer development.

The arrest at G2/M is regulated by the sequential activation and deactivation of CDK family proteins and Cyclin complexes, such as CDK1/CyclinB complex, which are associated with the entrance into mitosis, thereby, regulating the G2/M transition. The phosphorylation of Tyr15 of CDK1 suppresses activity of CDK1/CyclinB1 kinase complex [18]. In this study, the G2/M arrest induced by USP39 knockdown was accompanied with the suppression of CDK1/CyclinB1 activity. Besides, RT-PCR has been used to detect the Aurora B mRNA levels (Fig. 1d), and there were significant differences of the USP39 mRNA levels between shCon and shUSP39(S2) groups, which demonstrated that the effect of USP39 in MTC might not depend on the Aurora B.

Previous studies have reported that USP39 functions in pre-mRNA splicing [12]. Therefore, further investigations should determine whether USP39 is involved in splicing of CyclinB and CDK1, as well as other genes that are essential for cell cycle control.

## Conclusions

In conclusion, knockdown of USP39 by RNAi inhibited cell growth due to inactivation of the CDK1/CyclinB complex. USP39 may play a critical role in MTC malignant proliferation in vitro.
